# The Cause of the Suspension of the New York College of Dentistry

**Published:** 1869-08

**Authors:** 


*The Cause of the Suspension of the New York College of Dentistry*—In the June No. of the *Journal* our readers were informed of the suspension of this institution; the following account of the trial, taken from a New York daily paper, will explain the cause of the injunction being granted which closed its doors: "This was an action brought by the Attorney General, in the nature of a *quo warranto*, to forfeit the charter of the Dental College of New York on the ground of misbehavior of the trustees. The chief charges against them were: disregard of their own by-laws, attempting to alter those by-laws irregularly in order to cover up such irregularity, and giving diplomas to four students who were not, according to their regulations, entitled to such diplomas.

There were besides some charges that officers of the College had used the provisions of the College, made for the charitable treatment of the poor, for their own gain. This, however, was not an essential point in the case. The main charge was the issuing of the certificates or diplomas to the young men at the last graduation. The purpose of the founders of the College seems, from their earlier rules, to have been to very largely increase the scientific requirements of dentists. Their first rules required three year's study from their students, This was afterwards modified so as to permit office work to count as time of study, and thus to reduce the period of actual attendance at the College to two years or somewhat less. The charge is that, at the last examination, they passed some young men who had not filled even these easier requirements, and the regents of the University having refused to approve these diplomas, an effort was made, in a hurry and irregularly, to amend the by-laws to cover the case.

"The answer of the College is, that in so far as the faculty of the College did act, they acted on the representation of the dean, who has since failed to be re-elected, and is now the promoter of this attack ; that as matter of fact, the young men were well prepared, and passed excellent examinations; that the trustees did not, in fact, issue diplomas, and have done nothing illegal, whatever they may have intended to do, and that their intentions were perfectly good.

"The case came up on a motion before Judge Cardozo, and after argument, an injunction order was granted, and a receiver of the College appointed, as provisional remedies.

"A motion is now made for a reargument of the former motion and the vacation of the former orders, the defendant adding affidavits containing somewhat more full and technical denials of the charges, and the parties appeared by counsel before Judge Cardozo. Mr. Courtney, counsel for the plaintiff, commenced his argument, and continued for about ten minutes, when Judge Cardozo was called away from the bench. After waiting for his return some ten or fifteen minutes, the counsel after a short conversation, handed up their papers to the clerk and left the court-room."

